# Social deprivation and exposure to health promotion. A study of the distribution of health promotion resources to schools in England

**DOI:** 10.1186/1471-2458-10-473

**Published:** 2010-08-10

**Authors:** Corina M Chivu, Daniel D Reidpath

**Affiliations:** 1 Centre for Public Health Research Brunel University Uxbridge, Middlesex, UB83PH UK; 2 Professor of Population Health & Director of Public Health School of Medicine and Health Sciences Monash University Jalan Lagoon Selatan, Bandar Sunway, 46150, Selangor DE Malaysia

## Abstract

**Background:**

Area deprivation is a known determinant of health. It is also known that area deprivation is associated with lower impact health promotion. It is less well known, however, whether deprived areas are less responsive to health promotion, or whether they are less exposed. Using data from a national, school-based campaign to promote vaccination against the human papilloma virus (HPV), the relationship between area deprivation and exposure was examined.

**Methods:**

Taking advantage of a health promotion campaign to provide information to schools about HPV vaccination, a cross sectional study was conducted to examine the relationship between area level, social deprivation, and take-up of (i.e., exposure to) available health promotion material. The sample was 4,750 schools across England, including government maintained and independent schools. The relationship between area deprivation and exposure was examined using bi- and multivariate logistic regression.

**Results:**

It was found that schools in the least deprived quintile had 1.32 times the odds of requesting health promotion materials than schools in the most deprived areas (p = .01). This effect was independent of the school size, the type of school, and the geographic region.

**Conclusion:**

The relationship between area deprivation and the impact of health promotion may be due, at least in part, to differential levels of exposure. The study was limited in scope, pointing to the need for more research, but also points to potentially important policy implications.

## Background

The presence of an area level, social gradient in health behaviors and health outcomes has been commonly observed in the literature [1-3]. People who live in more socially deprived areas tend to experience worse health outcomes [4-8], and have a greater prevalence of behavioural risk factors [9,10], than those who live in less socially deprived areas. In the UK, for instance, people living in more deprived areas tend to have less healthy diets, higher rates of smoking, and lower levels of physical activity [11,12]. All of these behaviors are known risk factors for poor health outcomes. The international picture is somewhat similar [9,13-15]; although, the exact nature of the relationship between area deprivation and health has been found to vary by context [16].

Differences in the rates of health damaging behaviors, such as smoking, form at least a part of any explanation for variations in health outcomes between areas of different levels of deprivation. There is also an area level social gradient in the impact of health promotion efforts, whereby the impact of health promotion tends to be less in more deprived areas. For instance, people living in more deprived areas tend to have lower rates of smoking cessation [13], lower rates of childhood vaccination [17], and lower uptake rates of screening services [18].

Explaining the relationship between area deprivation and health remains an active field of research. Explaining the relationship between area deprivation and the impact of health promotion activities has been the focus of far less research. Broadly, however, two kinds of explanation present themselves. The first kind of explanation relates to the receptiveness of people living in more deprived areas to health promotion messages. People in more deprived areas may "choose" to ignore the health promotion messages more than those in less deprived areas [19]. A second kind of explanation is that people living in more deprived areas are just as receptive to health promotion messages, but they are less likely to be exposed to health promotion messages than their counterparts in less deprived areas. It is this latter kind of explanation that is explored in this paper.

In June 2007 the UK Department of Health (DH) accepted (in principle) the recommendation of the Joint Committee on Vaccination and Immunization to introduce a national, school-based, human papilloma virus (HPV) vaccination campaign, targeting girls [20]. The long-term goal of the campaign was to reduce the incidence of cervical cancer [21]. In England the vaccination campaign commenced in September 2008 - the beginning of the school year [22]. The vaccination was provided free of charge and on a voluntary basis, initially to 12 and 13 year old girls in schools, but the program was to be expanded to capture older female students (up to 18 years) who would otherwise miss out. In June 2008, prior to the commencement of the vaccination campaign, the Royal Society for Public Health (RSPH) developed and distributed to schools a series of health education resources on the HPV vaccine and cervical cancer [23]. The resources were developed in response to the government initiative, and were designed to support the vaccination campaign. The RSPH anticipated that, by providing schools with HPV related, health education resources which were integrated with the regular school curriculum, students (and through them, parents) would be made aware of the risk of HPV, the benefits of vaccination, and ultimately choose to have the vaccination [23].

The distribution of the educational resources to schools by RSPH created the conditions of a "natural experiment" allowing us to examine the effect of area level deprivation on the exposure to health promotion messages. It was a particularly pertinent campaign on which to focus, because cervical cancer is known to be unequally distributed in England, with the most deprived areas having twice the incidence of cervical cancer as the least deprived areas [24]. By analysing the uptake and distribution of the educational resources (i.e., the reach of health promotion), it was possible to measure the extent to which deprivation affected exposure to health promotion messages. If deprivation was associated with a lack of exposure, then one would anticipate that schools in more deprived areas would be less likely to receive HPV educational resources than would schools in less deprived areas.

## Methods

In March 2008, a letter was mailed by the RSPH to 5,715 schools across the UK inviting them to receive the HPV health education resources. The schools were drawn from a comprehensive database of UK secondary schools provided to the RSPH by a third party. Of the schools to be sent a letter, the majority (4,750, or 83.6%) was located in England, and it was these schools that formed the sample for the present analysis. The remaining 965 schools in Scotland, Wales and Northern Ireland were not included in the analysis, because of difficulties in matching the schools with equivalent measures of area deprivation. For each school additional limited data were available about the type and size of the school.

### Measures

The **outcome **measure, exposure to health promotion materials, was operationalised in terms of the take-up by schools of the RSPH educational resources. The requests for materials were recorded by the RSPH for all 4,750 of the schools included in the initial mail-out in England. The cut-off date for recording the requests was 29 July, 2008.

The **independent **variable, area deprivation, was measured using the Index of Multiple Deprivation 2007 (IMD) for England [25]. The IMD combines 37 indicators related to a range of economic, social and housing issues, into a single area deprivation score with the most deprived areas having the highest scores. The IMD has been used extensively to examine the relationship between area deprivation and health in England [11,26]. In the present analysis, the geographical location of each school was determined using the postcode to which the initial letters were sent. Each postcode was associated with an IMD score using the online GeoConvert facility [27]. The IMD scores for all the schools in the sample were then divided into quintiles of deprivation.

**The covariates **included in the analyses were school size, school type, and the geographic region within England where the school was located. School size was a dichotomous variable capturing the smallest 20% of schools, in terms of the size of the student body, versus the rest. The decision to dichotomize this variable and choice of cut-point was determined empirically during data exploration and cleaning. Schools type was a dichotomous variable capturing government maintained schools (i.e., schools receiving state aid) and independent schools. The geographical region was based on the 9 government office regions (GORs) of England. From North to South, these regions were the: North West, North East, Yorkshire and the Humber, West Midlands, East Midlands, East, London, South East, and South West. Matching a postcode to a GOR was, again, performed using the online GeoConvert facility [27].

### Data Analysis

Logistic regression was used to examine the relationship between area deprivation and the take-up of the HPV health educational resources. The approach involved standard progressive modeling whereby each of the possible unadjusted effects for the covariates was also examined. Then the effect of area deprivation adjusting for school size, school type, and region was estimated in a multivariate logistic model. When reporting the results of the logistic regression, the base category of the covariates was selected so that improvement in the take-up of the HPV educational resources was reflected as an odds ratio greater than 1.

The protocol for the study was approved through the formal ethics review processes of Brunel University.

## Results

Of the 4,750 schools sent a letter about the HPV health educational resources, 1,327 schools (27.9%) requested a copy. Table 1 shows a break down of the characteristics of schools that did and did not request the health education resources.

**Table 1:** The request for educational resources broken down by IMD, school size, school type, and geographic region.

	Received		Not Received		Unadjusted OR (95% CI)
	%	N	%	N	
**IMD quintiles**					
1 (Least Deprived)	31.7	301	68.3	648	1.38 (1.13-1.69)
2	30	284	70	664	1.27 (1.04-1.56)
3	28.4	269	71.6	679	1.18 (0.96-1.45)
4	24.7	235	75.3	715	0.98 (0.8-1.21)
5 (Most Deprived)	25.1	238	74.9	709	1 (Base)
**School size**					
Smallest 20%	17.3	167	82.7	798	1 (Base)
Remaining 80%	30.6	1160	69.4	2625	2.11 (1.76-2.53)
**School type**					
Independent	22.5	235	77.5	808	1 (Base)
Government Maintained	29.5	1092	70.5	2615	1.44 (1.22-1.69)
**GOR**					
London	19.9	145	80.1	584	1 (Base)
North West	29.1	180	70.9	438	1.66 (1.29-2.13)
North East	20.9	53	79.1	201	1.06 (0.75-1.51)
Yorkshire and the Humber	29.8	131	70.2	308	1.71 (1.30-2.25)
West Midlands	27.3	142	72.7	379	1.51 (1.16-1.97)
East Midlands	32.8	104	67.2	213	1.97 (1.46-2.65)
East	33.7	173	66.3	340	2.05 (1.58-2.65)
South East	27.4	240	72.6	635	1.52 (1.20-1.93)
South West	33	158	67	321	1.98 (1.52-2.57)

Unadjusted odds ratios and 95% confidence intervals are also shown.

The take-up of the educational resources varied across the quintiles of deprivation. Schools located in the most (and second most) deprived areas had take-up rates around 25%, while the schools located in the least deprived quintile had take-up rates around 32%. A test for trend showed a significant increase in the take-up of the educational resources as deprivation decreased (z = -3.99, p = .000). School size was significantly associated with the take-up of the HPV educational resources, with odds of larger schools taking up the educational resources more than twice that of the smallest 20% of schools (OR = 2.11, 95%CI: 1.76-2.53). Significantly more government maintained schools requested materials (32%) than independent schools (27%) (OR = 1.44, 95%CI: 1.22-1.69).

There was substantial regional variation in the take-up of the educational materials. London and the North East had the lowest take-up rates (~20%). The West Midlands and the South East had take-up rates around 27%. The North West and Yorkshire and the Humber had take-up rates around 30%. The East Midlands, the South West, and the East had take-up rates around 33%. All regions, with the exception of the North East had take-up rates significantly higher than that of London, with odds ratios ranging from 1.5 up to 2.1.

The multivariate logistic model shows the association between area deprivation and the take-up of the educational materials after adjusting for school size, school type, and the regional location of the school (Table 2).

**Table 2:** The adjusted odds ratios for requesting educational resources including 95% confidence intervals and approximate p-values.

Variables	Adjusted odds ratio	95% CI	p-value
**IMD quintiles**			
1 (Least Deprived)	1.32	1.06-1.63	.011
2	1.25	1.01-1.54	.037
3	1.16	0.94-1.43	.166
4	0.95	0.77-1.17	.608
5 (Most Deprived)	1 (Base)		
**School size**			
Smallest 20%	1 (Base)		
Remaining 80%	2.18	1.75-2.7	.000
**School type**			
Independent	1 (Base)		
Government Maintained	0.97	0.80-1.18	.790
**GOR**			
London	1 (Base)		
North West	1.57	1.21-2.02	.001
North East	1.02	0.71-1.46	.913
Yorkshire and the Humber	1.63	1.24-2.15	.001
West Midlands	1.45	1.11-1.9	.006
East Midlands	1.83	1.36-2.48	.000
East	1.88	1.44-2.45	.000
South East	1.45	1.14-1.85	.003
South West	1.9	1.45-2.48	.000

After adjusting for the covariates, there remained a significant association between area deprivation and the take-up of the HPV educational resources. Schools in the least deprived areas had odds 1.32 times greater than schools in the most deprived areas of requesting the teaching resources (p = .01). After adjustment, there remained a significant trend, with decreasing levels of deprivation associated with increasing take-up rates of the educational resources (p = .001) [28]. The interpretation of the covariates, post adjustment, was generally the same as the interpretation prior to adjustment. Larger schools were significantly more likely to take-up the teaching resources than smaller schools (OR = 2.16, 95%CI: 1.74-2.68). Geographical region also remained significantly associated with the take-up of the educational resources - indeed there were only minor variations in the odds ratios pre- and post adjustment. The major variation in the results was that the type of school (government maintained or independent) was no longer significantly associated with take-up post adjustment.

## Discussion

In 1986 the *Ottawa Charter for Health Promotion *detailed the basic goals and objectives for health promotion [29]. One of the goals was to achieve equity in health, and one of the strategies for doing this was to ensure equality of access to information. The motivation behind the strategy was the view that people can make healthier choices (in this case choose to be vaccinated), if they have appropriate information [30]. It is this issue of equality of access to information that lies at the heart of the present study. In an environment in which it is known that the most deprived areas have twice the incidence of cervical cancer as the least deprived areas [24], the most deprived and the least deprived areas should have, at a minimum, the same level of exposure to health relevant information. Indeed, as a matter of policy, it may be preferred for those areas with the highest incidence to have an even greater exposure to relevant information than those less deprived area with a lower incidence.

As anticipated, schools in the least deprived areas were significantly more likely to request the educational resources than schools in the most deprived areas; furthermore, this association held even after adjusting for school type, size and geographical region. Although the relationship between area deprivation and request for teaching resources was not particularly strong (OR = 1.32), given the association between deprivation and incidence of cervical cancer, any significant trend in exposure to health promotion material in the wrong direction is cause for concern and further investigation.

One explanation for the finding that less deprived areas had a higher take up rate of the educational resources may relate to the "inverse equity hypothesis" [31,32]. According to this hypothesis, higher socioeconomic status groups pick up interventions quicker than lower socioeconomic status groups. This increases the health differences between the groups in the short term. However, with the passage of time, the lower socioeconomic groups begin to pick up the intervention, which then reduces the health differences between the groups. It may be that schools in less deprived areas are better placed to take advantage of the freely available educational resources, explaining their quicker take up. Assuming the hypothesis is correct, and given continued availability of the materials [33], the difference in the take up rate may reduce over time.

In addition to the modest deprivation effect, there was a regional effect, with schools from some regions (the East and South West) having almost twice the odds of requesting the education resources as schools in London or the North East (with the highest percentage of small deprivation areas in the most deprived area quintile [25]). There was also a school size effect, with the smallest 20% of schools half as likely to request the materials as the larger schools.

It is tempting to speculate why factors such as area, school size, or indeed school type might be significantly associated with the take-up of the health promotion resources. One might speculate for example that religion could underpin school size or school type effects; perhaps with more religious (and possibly conservative) schools less supportive of the HPV vaccination program. Alternatively, it might be that smaller schools, with fewer staff, simply lack the capacity to take advantage of support offered through external initiatives. Unfortunately, the data are such that none of these questions can be adequately disentangled, and any response remains purely speculative.

There are two important limitations to the findings. The first limitation relates to the operationalisation of exposure to health promotions materials. The relationship between an individual's exposure to the health promotion materials and a school's request for materials is essentially unknown in this study. It may be that schools that did not request the materials from the RSPH but obtained the materials through a secondary source. Nor is it sufficient simply for school to request the materials, they have to be integrated into the curriculum which could be constrained by competition with other topics [34], school policy or local culture [35]. The extent to which materials are integrated into the curriculum will also affect individual's exposure. Nonetheless it is reasonable to assume that, on average, students in schools requesting the materials had a higher exposure to those materials than students in schools that did not request the materials. The second limitation relates to the design. As a "natural experiment" there was no control, and a range of unmeasured (and unknown) possible confounders, making any inference about a causal relationship impossible.

There is, prima facie, a third limitation, which on reflection is unfounded. The "third limitation" is that the area deprivation of a school does not reflect the area deprivation of the students within the school. This makes less sense when it is actually drawn out. The argument would be that, on average, students attending schools located in the most deprived areas are no more likely to live in most deprived areas than students attending schools in least deprived areas. It is true that students attending independent (privately funded) schools may travel considerable distances to attend school. Schools in receipt of government aid, however, tend to draw their student body from their local area. So the area level of deprivation of a government aid school is going to be similar (the data were in quintiles) to the area in which the students live. The adjustment in the analysis for school type was particularly pertinent to this argument; and even after adjustment, students attending schools in more deprived areas were significantly less likely to be exposed to the health promotion material.

Notwithstanding the limitations, the findings support the central idea that more deprived areas are likely to have a lower exposure to health promotion messages than less deprived areas. The effect is not strong, but the accumulation of weak effects over time, can have important ramifications for population health [36], and for the disparity in the health of more and less socially deprived groups. The interaction between the level of exposure, the level of area deprivation, and the individual response to health promotion messages would be a fruitful line of future inquiry.

## Conclusions

There was a social gradient associated with schools' response to the opportunity to receive educational resources supporting the HPV vaccination campaign. Schools in the most deprived areas were less likely to request materials than schools in less deprived areas. This was independent of other significant associations, such as school size and geographical region. This has important implication for the level of exposure to health promotion messages that people from more and less deprived areas are likely to experience.

## Competing interests

The authors declare that they have no competing interests.

## Authors' contributions

CC conceived the study, analysed and interpreted the data, drafted and edited the manuscript. DR contributed to the conception of the study, provided assistance with the analysis and interpretation of the data and edited the manuscript. Both authors read and approved the final manuscript.

## Pre-publication history

The pre-publication history for this paper can be accessed here:

http://www.biomedcentral.com/1471-2458/10/473/prepub

## References

[B1] Marmot M Wilkinson RG Social Determinants of Health 2005 2 USA: Oxford University Press

[B2] Siegrist J Marmot M Social Inequalities in Health: New Evidence and Policy Implications 2006 1 USA: Oxford University Press

[B3] Romeri E Baker A Griffiths C Mortality by deprivation and cause of death in England and Wales, 1999-2003 Health Statistics Quarterly/Office for National Statistics 2006 32 19 34 17165467

[B4] Fukuda Y Nakamura K Takano T Higher mortality in areas of lower socioeconomic position measured by a single index of deprivation in Japan Public Health 2007 121 3 163 173 10.1016/j.puhe.2006.10.015 17222876

[B5] Graham DJ Stephens DA Decomposing the impact of deprivation on child pedestrian casualties in England Accident; Analysis and Prevention 2008 40 4 1351 1364 10.1016/j.aap.2008.02.006 18606266

[B6] Daponte-Codina A Bolivar-Munoz J Toro-Cardenas S Ocana-Riola R Benach-Rovira J Navarro-Lopez V Area deprivation and trends in inequalities in self-rated health in Spain, 1987--2001 Scandinavian Journal of Public Health 2008 36 5 504 515 10.1177/1403494807088454 18567655

[B7] Scanlon PH Carter SC Foy C Husband RFA Abbas J Bachmann MO Diabetic retinopathy and socioeconomic deprivation in Gloucestershire Journal of Medical Screening 2008 15 3 118 121 10.1258/jms.2008.008013 18927093

[B8] Testi A Ivaldi E Material versus social deprivation and health: a case study of an urban area The European Journal of Health Economics: HEPAC: Health Economics in Prevention and Care 2009 10 3 323 328 19018580 10.1007/s10198-008-0136-z

[B9] Adams RJ Howard N Tucker G Appleton S Taylor AW Chittleborough C Effects of area deprivation on health risks and outcomes: a multilevel, cross-sectional, Australian population study International Journal of Public Health 2009 54 3 183 192 10.1007/s00038-009-7113-x 19214382

[B10] Ocaña-Riola R Saurina C Fernández-Ajuria A Lertxundi A Sánchez-Cantalejo C Saez M Area deprivation and mortality in the provincial capital cities of Andalusia and Catalonia (Spain) Journal of Epidemiology and Community Health 2008 62 2 147 152 10.1136/jech.2006.053280 18192603

[B11] Amuzu A Carsona C Watt HC Lawlor DA Ebrahim S Influence of area and individual lifecourse deprivation on health behaviours: findings from the British Women's Heart and Health Study European Journal of Cardiovascular Prevention and Rehabilitation 2009 16 2 169 173 10.1097/HJR.0b013e328325d64d 19242356

[B12] Gray L Leyland AH A multilevel analysis of diet and socio-economic status in Scotland: investigating the 'Glasgow effect' Public Health Nutrition 2009 12 9 1351 8 10.1017/S1368980008004047 19026094

[B13] Giskes K van Lenthe FJ Turrell G Brug J Mackenbach JP Smokers living in deprived areas are less likely to quit: a longitudinal follow-up Tobacco Control 2006 15 6 485 488 10.1136/tc.2006.015750 17130379 PMC2563671

[B14] Stimpson JP Nash AC Ju H Eschbach K Neighborhood Deprivation is associated with lower levels of serum carotenoids among adults participating in the Third National Health and Nutrition Examination Survey Journal of the American Dietetic Association 2007 107 11 1895 1902 10.1016/j.jada.2007.08.016 17964308

[B15] Utter J Scragg R Mhurchu CN Schaaf D At-home breakfast consumption among New Zealand children: associations with body mass index and related nutrition behaviors Journal of the American Dietetic Association 2007 107 4 570 576 10.1016/j.jada.2007.01.010 17383261

[B16] Ecob R Macintyre S Small area variations in health related behaviours; do these depend on the behaviour itself, its measurement, or on personal characteristics? Health & Place 2000 6 4 261 274 10.1016/s1353-8292(00)00008-3 11027952

[B17] Middleton E Comparison of social distribution of immunisation with measles, mumps, and rubella vaccine, England, 1991-2001 BMJ 2003 326 7394 854 854 10.1136/bmj.326.7394.854 12702619 PMC153473

[B18] Maheswaran R Pearson T Jordan H Black D Socioeconomic deprivation, travel distance, location of service, and uptake of breast cancer screening in North Derbyshire, UK Journal of Epidemiology and Community Health 2006 60 3 208 212 10.1136/jech.200X.038398 16476749 PMC2465550

[B19] Lynch JW Kaplan GA Salonen JT Why do poor people behave poorly? Variation in adult health behaviours and psychosocial characteristics by stages of the socioeconomic lifecourse Social Science & Medicine 1997 44 6 809 819 10.1016/s0277-9536(96)00191-8 9080564

[B20] Begum F Gates P Human Papillomavirus (HPV) Vaccine Uptake, Supply and Wastage Surveys 2008/09 http://docs.google.com/viewer?a=v&q=cache:YCDHb9uvSusJ:www.immunisation.nhs.uk/files/HPV_PCTUserGuide_HIP.pdf+http://nds.coi.gov.uk/environment/fullDetail.asp%3FReleaseID%3D293322%26NewsAreaID%3D2&hl=en&pid=bl&srcid=ADGEESj2i6wgbWqGZM7RUa3k2O4TetgtfYUTzp82c46HXdPCFQugNCmJrDCT75WhlC1VLYTQcKgCPF_RrnEYhEH0o51k1BZZmASoQricSDq5ZL0AFWcKDEwGFDfHCwlz_U2YgVv8niI&sig=AHIEtbTlcO2XVR3E3j8tMRAfFZcfhSajeA]

[B21] Castellsagué X Natural history and epidemiology of HPV infection and cervical cancer Gynecologic Oncology 2008 110 3 Suppl 2 S4 7 10.1016/j.ygyno.2008.07.045 18760711

[B22] NHS Choices: HPV-vaccination - Introduction http://www.nhs.uk/Conditions/HPV-vaccination/Pages/Introduction.aspx

[B23] Royal Society for the Promotion of Health: HPV Teaching and Learning Resource http://www.rsph.org.uk/en/policy-and-projects/projects/hpv-programme/hpv-teaching-and-learning-resource.cfm

[B24] Shack L Jordan C Thomson CS Mak V Møller H Variation in incidence of breast, lung and cervical cancer and malignant melanoma of skin by socioeconomic group in England BMC Cancer 2008 8 271 10.1186/1471-2407-8-271 18822122 PMC2577116

[B25] Noble M McLennan D Wilkinson K Whitworth A Barnes H The English Indices of Deprivation 2007 2007 London

[B26] Chin RFM Neville BGR Peckham C Wade A Bedford H Scott RC Socioeconomic deprivation independent of ethnicity increases status epilepticus risk Epilepsia 2009 50 5 1022 1029 10.1111/j.1528-1167.2008.01796.x 19178565

[B27] CDU: Census Dissemination Unit Census 2001 http://geoconvert.mimas.ac.uk/

[B28] Selvin S Statistical Analysis of Epidemiologic Data 1996 2 USA: Oxford University Press

[B29] World Health Organization Ottawa Charter for Health Promotion 1986 WHO, Geneva

[B30] Department of Health (UK) Choosing Health: Making healthy choices easier 2004 DOH, London

[B31] Aurélio Peres M Simara Fernandes L Glazer Peres K Inequality of water fluoridation in Southern Brazil - the inverse equity hypothesis revisited Social Science & Medicine 2004 58 6 1181 1189 10.1016/s0277-9536(03)00289-2 14723912

[B32] Victora CG Vaughan JP Barros FC Silva AC Tomasi E Explaining trends in inequities: evidence from Brazilian child health studies Lancet 2000 356 9235 1093 1098 10.1016/S0140-6736(00)02741-0 11009159

[B33] Royal Society for Public Health - Projects - HPV programme http://www.rsph.org.uk/en/policy-and-projects/projects/hpv-programme/

[B34] Buston K Wight D Hart G Scott S Implementation of a teacher-delivered sex education programme: obstacles and facilitating factors Health Education Research 2002 17 1 59 72 10.1093/her/17.1.59 11888044

[B35] Schaalma HP Abraham C Rogers Gillmore M Kok G Sex Education as Health Promotion: What Does It Take? Archives of Sexual Behavior 2004 33 3 259 269 10.1023/B:ASEB.0000026625.65171.1d 15129044

[B36] Reidpath D Diamond MR Hartel G Glasziou P Improving interpretability: γ As an alternative to R2 as a measure of effect size Statistics in Medicine 2000 19 10 1295 1302 10.1002/(SICI)1097-0258(20000530)19:10<1295::AID-SIM493>3.0.CO;2-Z 10814978

